# Hemodynamic analysis of thrombosed intracranial aneurysms: a comparative correlation study

**DOI:** 10.1007/s10143-025-03566-2

**Published:** 2025-05-14

**Authors:** Zonghan Lyu, Andres Gudino, Carlos Dier, Elena Sagues, Ivonne Salinas, Gustavo Chiriboga, Shubhangi Setia, Navami Shenoy, Edgar A. Samaniego, Jingfeng Jiang

**Affiliations:** 1 Department of Biomedical Engineering, Michigan Technological University, H-STEM 339, 1400 Townsend Drive, Houghton, MI 49931 USA; 2 Center for Biocomputing and Digital Health, Institute of Computing and Cybernetics, Health Research Institute, Michigan Technological University, Houghton, MI 49931 USA; 3 Department of Neurology, University of Iowa, 200 Hawkins Dr., Iowa City, IA 52242 USA; 4 Department of Radiology, University of Iowa, Iowa City, IA 52242 USA; 5 Department of Neurosurgery, University of Iowa, Iowa City, IA 52242 USA

**Keywords:** Intra-saccular thrombosis, Hemodynamics, Wall shear stress divergence, Velocity informatics, Flow disturbance

## Abstract

A small fraction of intracranial aneurysms (IA) contains intrasaccular thrombosis (IST). This study explores the hemodynamic causes of IST formation in IAs. We performed computational hemodynamic analysis in 26 IAs: 13 thrombosed and 13 non-thrombosed. The IAs in the two cohorts were matching in size and anatomical location. The computational hemodynamic analysis used “patient-specific” IA geometries derived from 3D magnetic resonance imaging. A comprehensive hemodynamic analysis was conducted using commonly used hemodynamic metrics (e.g., wall shear stress [WSS] and its derivative), flow vortex analysis, and velocity informatics. We observed that more flow eddies and endothelial cell activation potential (ECAP) are present in thrombosed IAs. The flow eddies in thrombosed IAs change size and location over a cardiac cycle more significantly than those in non-thrombosed IAs. These two factors, coupled with more flow stagnation and positive WSS divergence in the thrombosed IAs, promoted thrombus formation in the thrombosed IA cohort.

**Supplementary Information:**

The online version contains supplementary material available at 10.1007/s10143-025-03566-2.

## Introduction

Spontaneous subarachnoid hemorrhage is the third most common type of stroke and is frequently related to intracranial aneurysmal (IA) rupture [1]. However, the biological aspects of aneurysm formation, growth, and rupture are poorly understood. Since only a small percentage (e.g., ~ 10%) of IAs form intrasaccular thrombosis [2] and the presence of intrasaccular thrombosis (IST) impacts IAs’ pathophysiology, [3] investigating why the IST forms only in a small fraction of IAs may provide information about the biology of brain aneurysms.

To this end, the primary objective of this study is to systematically investigate the role of local hemodynamics in IST formation. Using an anatomical location- and size-matched control cohort, we comprehensively investigated time-resolved aneurysmal hemodynamics in a cohort of thrombosed IAs. Traditional and several novel hemodynamic variables, including (normalized) wall shear stress (WSS) divergence, [4] aneurysmal flow vortex core analysis, [5, 6] and velocity-informatics, [7, 8] were correlated to IST. This paper provides a comprehensive hemodynamics study of a large cohort of IAs with IST.

## Methods and materials

### Patient cohort

After institutional review board approval, patients with IAs were imaged using high-resolution magnetic resonance imaging (HR-MRI) with a 3 T (MAGNETOM, Skyra, Siemens) at the University of Iowa, and they were analyzed. Inclusion criteria involved the presence of IAs underlying spontaneous IST. Cases with artifacts or bad-quality imaging were excluded. Informed consent was waived because this study only used de-identified imaging data.

### MR data acquisition

T1-weighted (T1) and T1 + Gadolinium (T1 + Gd) sequences were obtained, isotropically resampled, and registered following a previously described pipeline [9]. T1 + Gd images were acquired 5 min after administering 0.1 mmol/Kg gadolinium-based contrast agent (Gadavist, Bayer Pharmaceuticals, Whippany, NJ), following our institution protocol (Supplementary Table S1). Demographic information was retrieved from electronic medical records. This study was similarly approved by the Michigan Technological University (Houghton, MI, USA). Our human subject study protocol follows the US National Institutes of Health’s guidelines on research involving Human Subjects, equivalent to the Declaration of Helsinki.

### Three-dimensional aneurysm segmentation and morphological characteristics

**Fig. 1:**
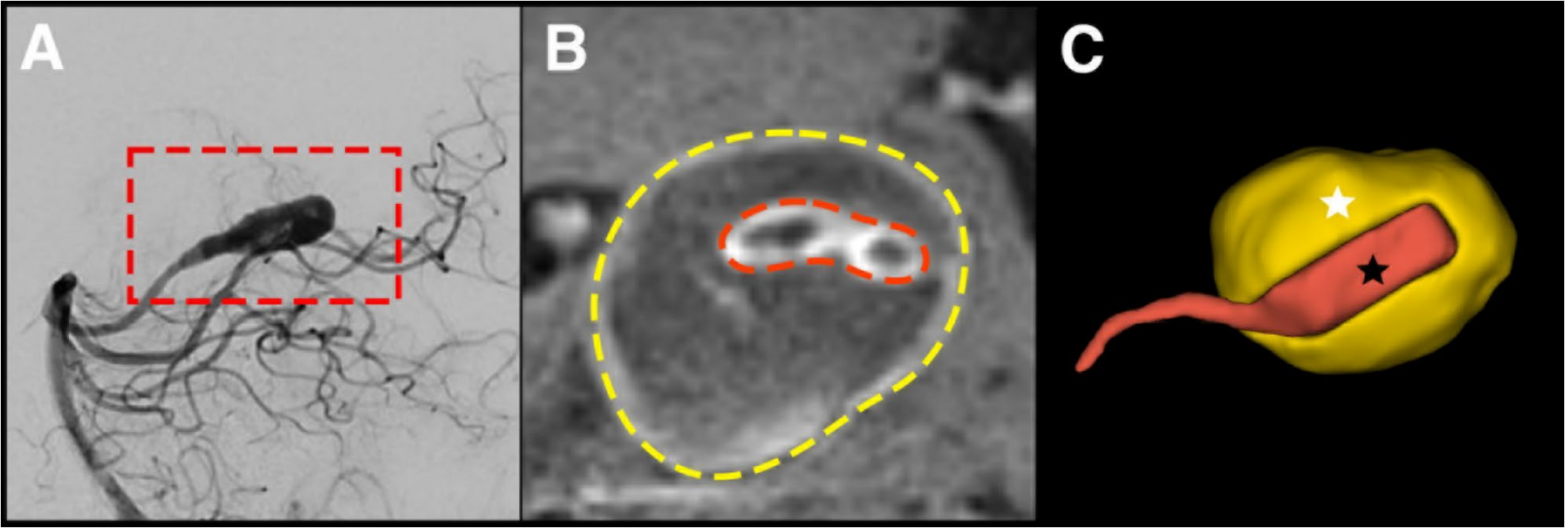
Schematic representation of three-dimensional segmentations. (**A**) Sagittal view of a digital subtraction angiography (DSA) showing a fusiform posterior cerebral artery aneurysm. (**B**) High-resolution magnetic resonance imaging (HR-MRI) shows the thrombosed segment (yellow dotted line) and luminal portion without thrombosis (red dotted line). (**C**) The boundaries of the clot and the parent artery are generated with a three-dimensional segmentation. The posterior cerebral artery is depicted in red (black star) and the thrombus in yellow (white star)

3D Slicer version 5.6.1 was used to perform three-dimensional (3D) segmentations [10]. Following a previously described pipeline, [9, 11] the 3D models included the non-thrombosed portion of the aneurysm, the thrombosed region, and the adjacent parent vessels, as visualized on T1 and T1 + Gd images. To accurately delineate the boundaries of the parent artery and thrombus, digital subtraction angiography (DSA) was used as a reference when available. In the absence of DSA, magnetic resonance angiography (MRA) or computed tomography angiography (CTA) was utilized (Fig. 1). A senior investigator (EAS) adjudicated the limits of aneurysms without thrombosis and thrombus boundaries, avoiding sampling surrounding structures. 3D models were generated for each case, and morphological measurements were retrieved. Aneurysm size was defined as the largest aneurysm diameter on both saccular and fusiform aneurysms [12, 13].

Table S2 of the Supplementary Materials provides definitions of aneurysm morphological parameters used in this study. Our prior publications provide more details of these morphological parameters [7, 14].

### Matching analysis

Hemodynamic metrics retrieved from thrombosed IAs were compared with equivalent hemodynamic parameters obtained from non-thrombosed IAs. For this purpose, a matching analysis was conducted between thrombosed and non-thrombosed IAs matched by aneurysmal size and location. 3D Slicer was used to generate 3D models following the previously described pipeline, and CFD simulations were similarly applied.

### CFD simulations

**Fig. 2:**
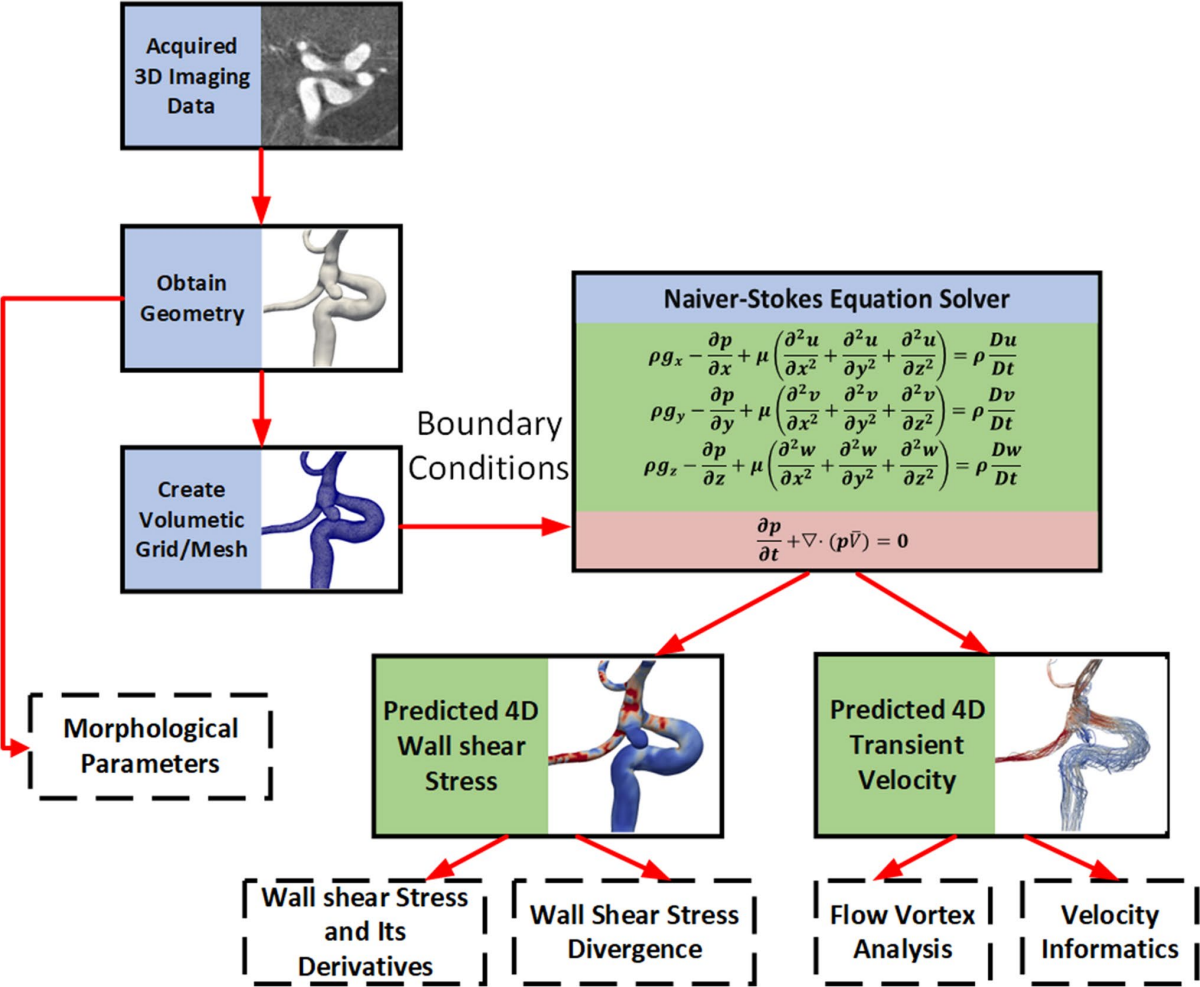
A diagram showing the workflow of our CFD simulations and post-processing of hemodynamic data and morphological parameters

CFD simulations were generated based on a previously described protocol (Fig. 2) [7, 8]. This workflow has been verified with both phase-contrast magnetic resonance angiography (PC-MRA) and ultrasound Doppler for aneurysmal flow [15–17].

“Patient-specific” vasculature geometries from 3D MRI using an open-source medical image processing named 3D Slicer package (version 21, https://www.slicer.org/, Kitware Inc. NY, USA) were obtained, similar to the procedures described in Section “Three-Dimensional Aneurysm Segmentation and Morphological Characteristics.”

Upon generating clean vessel geometries, cylindrical flow extensions with a minimum size of 10 times the vessel diameter were added to all inlets and outlets using the open-source Vascular Modelling Toolkit (VMTK) package (version 1.4, https://www.vmtk.org/). Unstructured 3D tetrahedral (volumetric) meshes with five boundary layers were generated with TetGen (Version 1.4.2). Models typically consist of a range of 0.5 million elements. Mesh sensitivity analysis was conducted to ensure appropriate mesh density, as demonstrated in our previous studies [7, 14].

Subsequently, a commercial CFD solver (Version 21, Fluent, Ansys Inc., PA, USA) was used to solve transient Navier–Stokes equations to compute blood flow velocity and WSS. Blood was modeled to be an incompressible, laminar, Newtonian fluid with a dynamic viscosity of 0.004 Pa·s and a mass density of 1040 kg/m^3^. Vessel walls, including the aneurysm wall, were assumed to be rigid, with a no-slip boundary condition. Depending on the anatomical locations of selected aneurysms, suited pulsatile flow (rate) waveforms were implemented as the inlet boundary condition. At the outlets, zero-pressure boundary conditions were prescribed during the CFD simulations. Four cardiac cycles were simulated at 1000 steps per period (0.001 s/timestep) with 20 constant interval data points.

### Evaluations of hemodynamic metrics

As shown in Fig. 2, upon the solution of Navier–Stokes equations, hemodynamic parameters in the following three categories were evaluated: (1) WSS variants, (2) WSS divergence parameters, and (3) flow vortex parameters.

At each phase of the cardiac cycle, a CFD-computed wall shear stress (WSS) vector $\tau =\left({\tau }_{x},{\tau }_{y},{\tau }_{z}\right)$ can be computed for each point on the aneurysmal sac. Hence, the time-average WSS (TWSS) for each point on the aneurysmal sac can be calculated as follows:

1 $$\mathrm{TWSS}=\frac{1}{T}\underset{0}{\overset{T}{\int }}\left|\tau \right|dt$$

where T is the duration of a simulated cardiac cycle and $\left|\tau \right|$ stands for the magnitude of the WSS vector. TWSS can be calculated for each point on the aneurysm sac. A spatial average of TWSS over the entire aneurysm sac becomes a singular value for each aneurysm, known as spatially and temporally averaged WSS (STAWSS).

The well-known oscillatory shear index (OSI) can be calculated for each point on the aneurysm sac as follows [18]:

2 $$\mathrm{OSI}=\frac{1}{2}\left(1-\frac{\underset{0}{\overset{T}{\int }}\tau dt}{\underset{0}{\overset{T}{\int }}\left|\tau \right|dt}\right)$$

The endothelial cell activation potential (ECAP) is linked to the potential of thrombus initiation [19] and can be computed for each point on the aneurysm sac as follows:

3 $$\mathrm{ECAP}=\frac{OSI}{TWSS}$$

The relative residence time (RRT) [20] is approximated by a combination of TWSS and OSI:

4 $$\mathrm{RRT}=\frac{1}{\left(1-2*OSI\right)*TWSS}$$

RRT is proportional to the relative duration that blood resides close to the wall.

We also normalized each WSS vector $\tau =\left({\tau }_{x},{\tau }_{y},{\tau }_{z}\right)$ to obtain a normalized WSS vector $\overline{\tau }=\left(\overline{{\tau }_{x}},\overline{{\tau }_{y}},\overline{{\tau }_{z}}\right)$ for each point of the aneurysm sac. Then, the normalized WSS divergence (NWSS_Div_) was expressed as follows:

5 $${\mathrm{NWSS}}_{\mathrm{Div}}=\frac{\partial \overline{{\tau }_{x}}}{\partial x}+\frac{\partial \overline{{\tau }_{y}}}{\partial y}+\frac{\partial \overline{{\tau }_{z}}}{\partial z}$$

where *x*, *y*, and *z* are the three-dimensional coordinates of the aneurysm sac. If NWSS_Div_ has a positive value, the net effect of WSS is to stretch the aneurysm sac; otherwise, WSS has a net effect of compressing the aneurysm sac.

The time-varying endothelial contraction and expansion for a given spatial location can be quantified using the Topological Shear Variation Index (TSVI) as follows, [21]

6 $$TVSI={\left\{\frac{1}{T}{\int }_{0}^{T}\left[NWS{S}_{Div}-\overline{{NWSS }_{Div}}\right]\right\}}^{1/2}$$

where T is a cardiac cycle, $NWS{S}_{Div}$ is an instantaneous normalized WSS divergence, and ${NWSS}_{Div}$ is the time-averaged normalized WSS divergence.

**Fig. 3:**
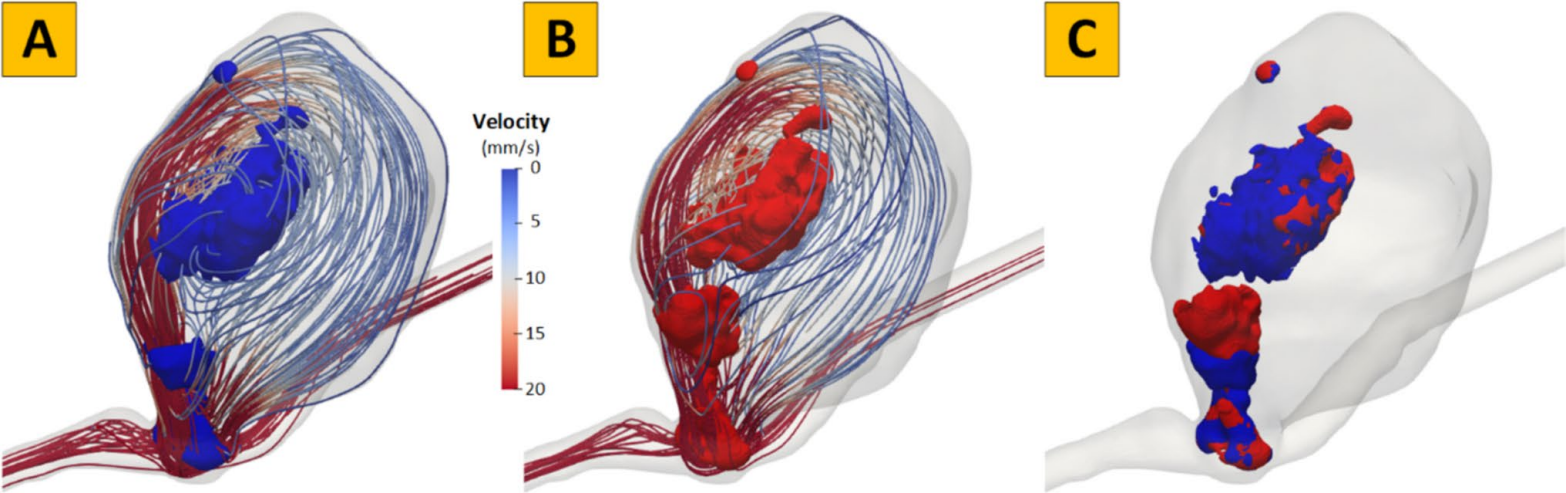
An example showing complex flow disturbance in an aneurysm. The degree of volume overlap (DVO) of flow vortex cores between two phases of a cardiac cycle: (**A**) the i^th^ time-step (blue), (**B**) the (i + 1)^th^ time-step (red), and (**C**) minimal flow vortex core overlaps indicating significant flow complexity. Velocity streamlines show the overall flow pattern at each phase

Swirling flow eddies (i.e., recirculation zones) are present in aneurysmal flow; typically, flow eddies shift, break, and merge in space over a cardiac cycle, as shown in Fig. 3. Using a published method, [5, 14, 22] we first segmented the flow vortex core regions for each phase of the cardiac cycle (see the blue and red surfaces in Figs. 2A and 2B). More details of the segmentation of the flow vortex core regions are included in Supplementary Materials Sect. 2. Then, we calculated the degree of overlap (DVO) defined as the overlap ratio between the flow vortex core regions at two adjacent phases (see Fig. 3C). Visualization of flow vortex cores over a cardiac cycle overlaid with time-resolved streamlines of the IA shown in Fig. 3 is provided in a video associated with this manuscript. More detailed information of vortex core analysis is provided in the Supplementary Materials (Sect. 3).

Hemodynamic parameters in the three categories (i.e., WSS variants, WSS divergence parameters, and flow vortex parameters) are summarized in Table 1.

**Table 1:** A summary of conventional hemodynamic parameters used in this study

Type	Parameter Name	Description
WSS- variants	SA ECAP	Spatially-averaged Endothelial cell activation potential, which is related to the potential of thrombus formation; calculated by Eq. (3) for each point and then spatially averaged over the entire aneurysm sac
	Max ECAP	Maximum of the Endothelial cell activation potential over the aneurysm sac
	Min ECAP	Minimum of the Endothelial cell activation potential over the aneurysm sac
	SA RRT	Spatially averaged relative residence time over the aneurysm sac; calculated by Eq. (4) for each point on the aneurysm sac and then spatially averaged over the entire aneurysm
	Max RRT	Maximum of relative residence time over the aneurysm sac
	Systole STAWSS	Spatio-temporally averaged wall shear stress at the peak systole
	Systole WSSMin	Minimum wall shear stress at the peak systole
	Systole WSSMax	Maximum wall shear stress at the peak systole
	SA OSI	Spatial average of the oscillatory shear index
	TA LSA 2	Temporally averaged low shear area less than 0.4 Pa
Flow Vortex	Systole TADVO	Temporally averaged degree of volume overlap during the systole phase
	Vortex V	Temporally averaged volume of vortex cores during the systole phase
	Num_core	Temporally averaged number of vortex cores during the systole phase
WSS Divergence	Mean-NWSS_Div_	Spatially and temporally averaged WSS divergence value; one value for each IA
	Mean-TVSI	Spatially averaged TVSI value; one value for each IA

### Velocity Informatics (VI) analysis

In addition to the above-mentioned three major categories of WSS and vortex parameters, we employed VI (magnitude and directional velocity-informatics; hereafter referred to as MVelocity-informatics and DVeloicity-informatics), [7, 23] as the fourth category for a comprehensive analysis of aneurysmal flow. VI assesses spatial patterns contained in the time-resolved velocity data, and many parameters correlate to characteristics of disordered aneurysmal hemodynamics. The extracted features include the Gray Level Co-occurrence Matrix (GLCM, 24 features), the Gray Level Run Length Matrix (GLRLM, 16 features), and the Gray Level Size Zone Matrix (GLSZM, 16 features). A brief introduction of VI variables is included in Supplementary Materials Sect. 4.

In this study, we focused on the peak systole phase for VI analysis, representing the period of maximal hemodynamic stress—characterized by peak wall shear stress and flow velocity magnitudes. This phase is physiologically relevant as these mechanical forces are crucial in endothelial cell signaling pathways and thrombus initiation mechanisms. The peak systolic velocity fields effectively capture the most energetic flow conditions, which predominantly determine particle residence times and near-wall flow patterns. Similarly, many conventional hemodynamic metrics listed in Table 1 were also related to the systole phase. This protocol is consistent with three of our prior studies [7, 14, 23].

### Statistical analysis

R version 4.3.3 was used for statistical analysis. Categorical variables are presented as N (%). Normally distributed variables are listed as mean $\pm$ standard deviation. Non-normally distributed variables are listed as median and interquartile range. Wilcoxon Rank Sum test was used to address differences between hemodynamic parameters and velocity informatics between thrombosed and non-thrombosed IAs. Due to the exploratory nature of this study, *p* values < 0.05 were considered statistically significant.

## Results

Through a search of our internal database, 44 thrombosed IAs were initially identified from our HR-MRI repository. Of these, 4 cases (9.1%) were excluded due to flow artifacts, and 2 cases (4.5%) due to poor image quality, leaving 38 thrombosed IAs for analysis. Among these, 21 (55.3%) were saccular, and 17 (44.7%) were fusiform. The most common location was the cavernous segment of the internal carotid artery (ICA), observed in 16 out of 38 cases (42.1%).

Given these 38 thrombosed IAs, only 14 thrombosed IAs could be matched regarding aneurysm size and location. Since only one thrombosed IA was located at the basilar artery, and the remaining thirteen were in the cavernous ICA, the basilar artery aneurysm was eliminated. Finally, 13 thrombosed ICA aneurysms were identified: 10 out of 13 (76.9%) thrombosed aneurysms in the cavernous ICA and 3 (23.1%) in the communicating segment of the ICA. These thrombosed IAs were matched with non-thrombosed IAs in identical locations (Supplementary Figure S2 and Table S2 in the Supplementary Materials Sect. 2).

### Comparison of conventional hemodynamic parameters

Fifteen commonly used hemodynamic parameters (Table 1) were compared between thrombosed and non-thrombosed IAs.

In Table 2, Wilcoxon Paired tests (MATLAB, Mathworks Inc., MA, USA) were calculated to determine whether each hemodynamic variable can discriminate against a thrombosed IA. Among all features, three (3) parameters from ECAP, WSS, and WSS divergence can discriminate against a thrombosed IA.

**Table 2:** A summary of selected conventional hemodynamic parameters used to compare thrombosed and non-thrombosed IA groups. Red font indicates statistical significance (*p*-value < 0.05)

Parameter	Cohort		*P*-value
	Thrombosed	Non-thrombosed	
OSI	0.02 ± 0.01	0.02 ± 0.01	0.735
Max ECAP	9.41 ± 16.32	2.64 ± 2.16	**0.048**
SA ECAP	0.12 ± 0.18	0.06 ± 0.07	0.068
Max RRT	230.22 ± 401.56	144.85 ± 197.86	0.340
SA RRT	3.44 ± 5.08	1.28 ± 1.02	0.080
Mean-TAWSS (Pa)	2.82 ± 1.81	3.34 ± 2.08	0.147
TA LSA (mm^2^)	62.12 ± 24.91	55.18 ± 25.68	0.127
STAWSS (Pa)	3.38 ± 2.26	4.09 ± 2.69	0.080
STAWSS_min (Pa)	0.06 ± 0.07	0.15 ± 0.23	**0.048**
STAWSS_max (Pa)	77.58 ± 52.97	100.68 ± 101.55	0.893
TADVO	0.52 ± 0.15	0.54 ± 0.17	0.542
Vortex_v (mm^3^)	905.43 ± 584.05	849.64 ± 506.01	0.356
num_core	6.67 ± 4.68	5.63 ± 3.65	0.662
Mean-NWSS_Div_	0.015 ± 0.013	0.006 ± 0.021	**0.049**
Mean-TSVI	0.127 ± 0.041	0.126 ± 0.026	0.99

### Comparison of VI parameters

We found a large number of parameters (34) that are statistically significant (*p*-value < 0.05) between thrombosed and non-thrombosed IAs (Table 3). More details of these parameters can be found elsewhere (https://pyradiomics.readthedocs.io/en/latest/features.html#) and described in Supplementary Materials Sect. 4.

**Table 3:** A summary of magnitude and direction VI parameters

Category	Parameter	Cohort		*P*-value
		Thrombosed	Non-thrombosed	
Magnitude VI	GLCM.Autocorrelation	1120.81 ± 1366.33	2786.28 ± 3741.48	** < 0.001**
	GLCM.ClusterTendency	1277.59 ± 1166.19	1924.34 ± 1647.77	**0.04**
	GLCM.DifferenceAverage	2.48 ± 2.39	3.54 ± 3.37	**0.006**
	GLCM.DifferenceEntropy	2.54 ± 0.93	3.00 ± 0.91	**0.002**
	GLCM.Id	0.60 ± 0.17	0.51 ± 0.15	**0.002**
	GLCM.Idm	0.56 ± 0.19	0.46 ± 0.17	**0.002**
	GLCM.Imc1	−0.48 ± 0.12	−0.43 ± 0.09	**0.003**
	GLCM.JointAverage	24.64 ± 14.87	40.88 ± 26.57	** < 0.001**
	GLCM.JointEnergy	0.02 ± 0.03	0.01 ± 0.01	**0.001**
	GLCM.JointEntropy	8.38 ± 1.93	9.49 ± 1.64	**0.003**
	GLCM.SumAverage	49.28 ± 29.73	81.75 ± 53.14	** < 0.001**
	GLCM.SumEntropy	6.37 ± 0.96	6.96 ± 0.75	**0.003**
	GLCM.SumSquares	329.05 ± 306.65	498.33 ± 440.33	**0.04**
	GLRLM.GLNN	0.03 ± 0.02	0.02 ± 0.01	**0.006**
	GLRLM.HGLRE	1300.53 ± 1330.01	2888.03 ± 3618.15	**0.001**
	GLRLM.LRE	11.01 ± 17.74	4.43 ± 4.64	**0.001**
	GLRLM.LRHGLE	2691.51 ± 1213.47	4809.15 ± 3748.69	**0.005**
	GLRLM.LRLGLE	0.98 ± 3.10	0.10 ± 0.24	** < 0.001**
	GLRLM.LGLRE	0.04 ± 0.05	0.02 ± 0.01	**0.002**
	GLRLM.RLN	207259.39 ± 118643.23	271661.34 ± 157264.05	**0.013**
	GLRLM.RLNN	0.57 ± 0.19	0.68 ± 0.14	**0.001**
	GLRLM.RunPercentage	0.62 ± 0.19	0.73 ± 0.15	**0.001**
	GLRLM.RunVariance	6.75 ± 12.47	2.18 ± 3.20	**0.001**
	GLRLM.SRE	0.77 ± 0.13	0.84 ± 0.09	**0.001**
	GLRLM.SRHGLE	1200.68 ± 1307.45	2693.87 ± 3535.08	**0.002**
	GLRLM.SRLGLE	0.02 ± 0.02	0.01 ± 0.01	**0.002**
	GLSZM.LAE	1708712.44 ± 4311351.24	388399.58 ± 969821.10	**0.002**
	GLSZM.LALGLE	214720.11 ± 735218.43	9282.70 ± 30906.16	** < 0.001**
	GLSZM.SZNU	20484.85 ± 10623.04	29025.85 ± 13038.06	**0.04**
	GLSZM.ZonePercentage	0.13 ± 0.13	0.18 ± 0.16	**0.006**
	GLSZM.ZoneVariance	1707650.03 ± 4308467.97	388098.02 ± 969033.74	**0.002**
Direction VI	GLCM.InverseVariance	0.11 ± 0.03	0.13 ± 0.03	**0.04**
	GLSZM.GLNN	0.003 ± 0.000	0.004 ± 0.000	**0.01**
	GLSZM.ZoneEntropy	10.49 ± 0.35	10.26 ± 0.51	**0.033**

## Discussions

This comprehensive analysis of a large cohort of thrombosed aneurysms determined that three interwoven “factors” contribute to clot formation in thrombosed IAs, as explained below.

ECAP reflects the interplay between TAWSS and OSI [24, 25], which has been linked to intraluminal thrombosis, mainly in aortic aneurysms. This association is thought to arise from low TAWSS promoting monocyte adhesion and high OSI contributing to atherosclerosis-prone behavior, collectively creating a thrombotic susceptibility marked by high ECAP. [25] However, the relationship between ECAP and thrombosis in IAs has not been fully explored. Notably, our CFD simulations found that maximum ECAP (Max ECAP; see Table 2) was significantly higher (*p* < 0.05) in thrombosed IAs compared to non-thrombosed aneurysms, offering a potential tool to address IST in IAs. Moreover, we found that spatially averaged RRT (SA-RRT; see Table 2) values are elevated (p-value = 0.08), while STAWSS values are also lower among thrombosed IAs (p-value = 0.08). These findings suggest that flow stagnation is more pronounced in thrombosed IAs than in non-thrombosed IAs. The slower blood flow may allow platelets and clotting factors to remain within the aneurysmal sac for extended periods (reflected in higher RRT), increasing their interaction with the aneurysmal wall. This prolonged interaction may promote platelet activation and aggregation, ultimately leading to thrombus formation.

We also found that thrombosed IAs exhibit slightly different flow patterns than non-thrombosed IAs. Recirculation zones are characterized by regions with the vortex motion of fluids [26], which has been associated with lower WSS [26]. Likewise, regions with lower WSS (see LSA in Table 2) have been proposed to generate a thrombosis-prone phenotype among IAs [27].

**Fig. 4:**
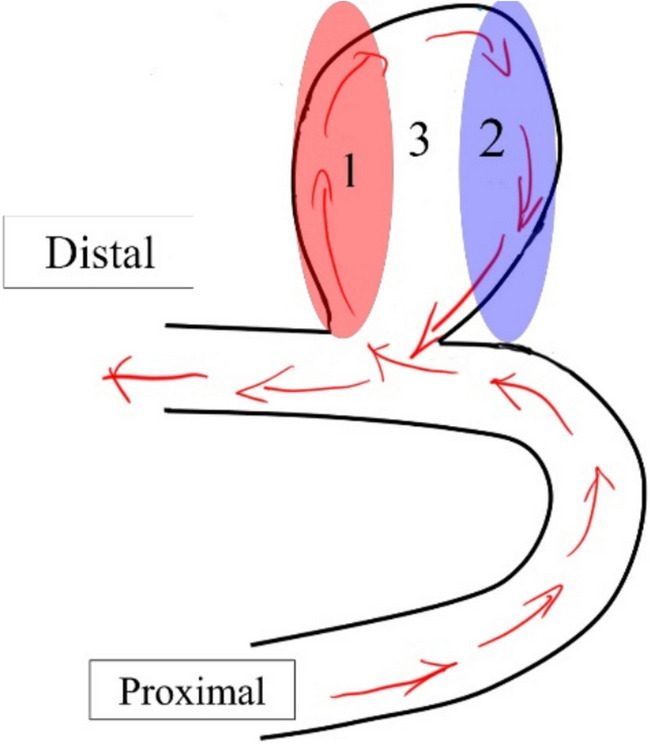
An illustrative example of a side-wall aneurysm. Zones 1–3 in the plot above stand for flow entering the aneurysm, flow leaving the aneurysm, and recirculation regions

Using human visual assessments, Cebral et al. established the association between the IA rupture risk and spatial flow complexity [28]. In contrast, VI provides a novel quantitative method for quantifying spatial flow complexity. As shown in Fig. 4, the aneurysmal flow in a typical side-wall aneurysm can be divided into three zones: (1) flow entering the IA, (2) flow leaving the IA, and (3) recirculation zones. It is important to note that flow eddies form within the aneurysmal sac (i.e., Zone 3) due to disturbed or disordered blood flow caused by the irregular geometry of the aneurysm and the incoming velocity jet entering it.

VI parameters in combination can depict heterogeneous disordered flow distributions among the aneurysm sac, as discussed in a recent publication [8]; that is why VI is also known as spatial pattern analysis of velocity patterns. The VI analysis is useful since the disordered aneurysmal flow plays a crucial role in IST within the IA sac. For example, the parameter GLRLM.HGLRE measures the distribution of higher velocities within the aneurysm dome. Thus, elevated GLRLM.HGLRE values observed in thrombosed IAs indicate the presence of stronger velocity jets entering the aneurysm domes (i.e., Zone 1). These stronger jets are conceptually associated with generating more disordered flow and larger eddies. The presence of large flow eddies within the thrombosed IA sacs (i.e., Zone 3) is further supported by several VI parameters, including higher GLRLM.GLNN, GLCM.Id, and GLCM.JointEnergy values, along with lower GLCM.Autocorrelation values. These observations collectively highlight the unique hemodynamic environment in thrombosed IAs, i.e., more complex recirculation zone(s) induced by stronger velocity jet(s) entering the IAs.

It is also important to note that in thrombosed IAs, small GLSZM.LAE values (see Table 3) suggest that low-velocity regions are relatively small. This observation is consistent with the results of flow-swirling vortex cores shown in Table 2, i.e., the higher number of vortex cores in thrombosed IAs. Also, the TADVO_std is larger in the thrombosed IAs than in the non-thrombosed IAs (not shown in Table 2), indicating that flow eddies shift and change their location over a cardiac cycle more significantly in the thrombosed IAs. During the cardiac cycle, many activated platelets may be trapped within swirling aneurysmal eddies. As the eddies change size and split over the cardiac cycle, they are transported to the vascular wall [29], promoting clot formation and causing the the degradation of structural support [30].

It is worth noting that determining the vortex core regions relies on a threshold (see Sect. 3 of the Supplementary Materials). Although the size of the vortex core regions generally increases as the threshold decreases, we verified that our results (Table 2) were stable when we changed this threshold from 0.15 to 0.3 with a 0.05 increment.

Third, as shown in Table 2, the positivity of NWSS_Div_ is higher in thrombosed IAs. According to the pioneering work of Mazzi et al. [21], normalized WSS (NWSS) can serve as a surrogate marker for near-arterial wall motion. Positive and negative NWSS_Div_ values correspond to elongational and compressive blood flow. Elongational flow, in particular, is a key mechanism for activating von Willebrand factor (vWF), a protein that plays a critical role in blood clotting [31]. Based on this, we hypothesize that thrombosed IAs may have a higher concentration of activated platelets near the aneurysmal wall, contributing to thrombus formation. However, the mean TVSI values [21] (see Table 2) did not show differences between the thrombosed and non-thrombosed groups, suggesting that the temporal variations of WSS divergence play a less important role.

We propose that the combination of the three factors outlined above contributes to the initiation of clot formation in certain IAs. Specifically, the complex flow patterns observed in thrombosed IAs (Fig. 3) promote platelet activation, [32–34] particularly through the second and third factors, and the stagnated flow within these aneurysms allows activated platelets to remain longer, fostering extensive thrombus formation. Our findings also indicate that VI parameters are effective in distinguishing thrombosed from non-thrombosed IAs by quantifying flow disturbances based on gross aneurysmal flow patterns. This approach has demonstrated predictive value in assessing rupture risk in IAs and growth status in abdominal aortic aneurysms [7, 23]. Moreover, in unruptured IAs, IST may either stabilize the aneurysm or accelerate its progression toward rupture. Understanding the hemodynamic mechanisms underlying IST formation is critical for elucidating why different types of thrombi (e.g., stable versus unstable) accumulate over time. Differentiating these thrombus types and exploring their hemodynamic causes remain important directions for future research. Additionally, CFD analysis has provided valuable tools to address the recanalization of previously treated IAs [35]. For example, high WSS has been recognized in aneurysm regions that were recanalized following coil embolization [36]. Therefore, expanding our knowledge of the hemodynamic nature of IST by offering new CFD metrics, including ECAP or detailed information about flow through velocity informatics, could be a potential tool in assessing IAs.

This study has several limitations. Although it represents the largest cohort of thrombosed IAs studied to date, the sample size remains relatively small. Furthermore, longitudinal HR-MRI imaging to assess thrombus progression or aneurysm rupture was unavailable, as most patients in our cohort underwent endovascular treatment. Additionally, the computational analysis used in this study is highly complex and not yet suitable for clinical application. To address this, we are actively working on automating the analysis to develop practical and clinically applicable parameters for predicting aneurysm thrombosis. Moreover, computational hemodynamic studies have largely adopted one of two outlet boundary condition frameworks: (1) variants of Murray’s law (e.g., [37–39]) and (2) zero-pressure outlet boundary conditions (e.g. [40, 41],). Recent studies indicate significant discrepancies in hemodynamic predictions depending on the choice of boundary conditions [42–44]. In this study, our primary goal was to investigate differences between thrombosed and non-thrombosed IAs under consistent boundary conditions. However, our findings suggest that boundary conditions may substantially influence the CFD results and the discernibility of hemodynamic differences between thrombosed and non-thrombosed IAs. The interplay of these factors warrants further investigation, which will be addressed in future work.

## Conclusions

The flow patterns described in this study suggest that thrombosed IAs are characterized by versatile shear layers and stronger elongational flow, which may enhance platelet activation. When coupled with the more stagnated flow observed in these aneurysms, the potential for clot formation is significantly increased. The ultimate objective of this and future research is to identify computational hemodynamic patterns that can reliably predict aneurysm thrombosis. Such insights could enhance our understanding of the underlying biology of IAs and inform the development of targeted medical interventions aimed at inducing controlled thrombosis in brain aneurysms.

## Supplementary Information

Below is the link to the electronic supplementary material.

Supplementary file1 (DOCX 1129 KB)

Supplementary file2 (AVI 637 KB)

## Data Availability

No datasets were generated or analysed during the current study.
