# Supplementary material for: Hemodynamic analysis of thrombosed intracranial aneurysms: a comparative correlation study
Source: Neurosurg Rev. 2025 May 14;48(1):417. doi: 10.1007/s10143-025-03566-2 (PMC12078405; doi:10.1007/s10143-025-03566-2)
Supplement: Supplementary file 1 — Supplementary file1 (DOCX 1129 KB) [file 10143_2025_3566_MOESM1_ESM.docx]

**Supplementary Materials to** Manuscript entitled “Hemodynamic Analysis of Thrombosed Intracranial Aneurysms: A Comparative Correlation Study”

**Section 1 - Patient Cohort: Image acquisition protocol**

As discussed in the Methods and Materials Section, T1-weighted (T1) and T1+Gadolinium (T1+Gd) sequences were acquired for all patients using the setup outlined in Table S1.

**Supplementary Table S1: 3T Siemens high-resolution magnetic resonance imaging protocol.**

|  | **SAG 3D T1 SPACE FS** |
| --- | --- |
| TR (msec) | 900 |
| TE (msec) | 15 |
| Flip angle (deg) | variable |
| Bandwidth (Hz/pixel) | 446 |
| FOV (mm) | 200 x 200 |
| Matrix (mm) | 320 x 320 |
| Voxel size (mm) | 0.6 x 0.6 x 0.6 |
| Slice thickness, mm | 0.63 |
| Turbo Factor | 52 |
| Acquisition time (min) | 3:29 |

**Section 2 – Aneurysm Geometrical Characteristics and Definitions**

The geometrical characteristics of selected in thrombosed and non-thrombosed groups are provided in Table S2 below.

**Supplementary Table S2:** Thrombosed and non-thrombosed matched groups. No statistical differences exist between the two cohorts.

| Group | Location | Height (mm) | Surface Area (mm^2^) | Volume (mm^3^) | Ostium Area (mm^2^) | Aspect  Ratio |
| --- | --- | --- | --- | --- | --- | --- |
| Thrombosed | 13 ICA | 23.5 $\pm$ 8.5 | 1545.8 $\pm$ 857.4 | 6307.2 $\pm$ 4515.8 | 72.1 $\pm$ 66.2 | 3.3 $\pm$ 1.4 |
| Non-thrombosed | 13 ICA | 20.9 $\pm$ 7.2 | 1392.9 $\pm$ 808.3 | 5391.7 $\pm$ 4202.5 | 43.8 $\pm$ 5.2 | 2.8 $\pm$ 1.0 |

The morphological parameters used in this study are described in Table S2 below. Our prior publications provide more details. [1, 2]

**Table S3**: A summary of morphological parameters used to characterize IAs.

| Parameter | Description |
| --- | --- |
| Aneurysm Volume | Volume of the aneurysm |
| Aneurysm Height | Height of the aneurysm |
| Sac Max Width | Maximum width of the aneurysm sac |
| Size Ratio Height | The size ratio between aneurysm height and parental artery diameter |
| Size Ratio Width | The size ratio between aneurysm width and parental artery diameter |
| Aspect Ratio Star | An aspect ratio of the intracranial aneurysm |
| Vessel Diameter | Diameter of the parental vessel connected to the aneurysm |
| Ostium Minimum | The maximal ostium diameter |
| Ostium Maximum | The minimal ostium diameter |
| Aneurysm Area | Area of the aneurysm |
| Ostium Area | Area of the ostium |

**Section 3 - Vortex core analysis**

A computational approach utilizing informational entropy was employed to determine the spatially varying direction of the velocity field for identifying flow vortices. The 3D angular space of the velocity field was first partitioned into 360 equal-area bins, forming conical regions that connect the center of a unit sphere to surface patches. Each velocity vector was assigned to a corresponding patch if it fell within the associated cone. To evaluate the local flow direction $x\in\{x_{1},x_{2},x_{3},\ldots,x_{n}\}$ within the velocity field $X$, the probability $p\left( x_{i} \right)$ of each direction was computed and used to determine Shannon’s entropy.

$$H\left( X \right)= -\sum_{x_{i}\in X} p\left( x_{i} \right){log}_{2}p\left( x_{i} \right)$$

$$NE\left( X \right)= \frac{H(X)}{{log}_{2}(N)}$$

Since normalized entropy (NE) cannot distinguish vortices from Brownian motion, the $\lambda_{2}$​ method was incorporated. The velocity gradient tensor was decomposed into strain-rate ($S$) and spin ($\Omega$) components. Vortex cores were identified where $S^{2}+\Omega^{2}$ had two negative eigenvalues ($\lambda_{1}>\lambda_{2}>\lambda_{3}$​). The dot product between the velocity vector and eigenvector measured directional alignment, with 0 indicating co-alignment and 1 indicating orthogonality.

$$S= \frac{\nabla\vec{v}+{\nabla\vec{v}}^{T}}{2}, \Omega= \frac{\nabla\vec{v}-{\nabla\vec{v}}^{T}}{2}$$

To refine vortex detection, this alignment factor $du(\theta)$ was multiplied by NE, reducing false positives from Brownian motion.

$$du\left( \theta\right)=\left\| \vec{v} \right\|\cdot\lambda_{2}$$

This combined method (CM) was applied across all voxels in the IA dome for each cardiac cycle. Vortex regions were extracted using the marching cubes algorithm, with a threshold CM>0.3. Small regions (<0.5 mm³) were excluded to prevent misidentification.

$$CM\left( X \right)=NE(X)\times du\left( \theta\right)$$

A video illustrating the vortex transition through one cardiac cycle from a sample case is also included.

**Section 4 - Velocity informatics**

This study employs the velocity-informatics technique to quantify blood flow characteristics using spatial patterns, as proposed in the previous publication [3].


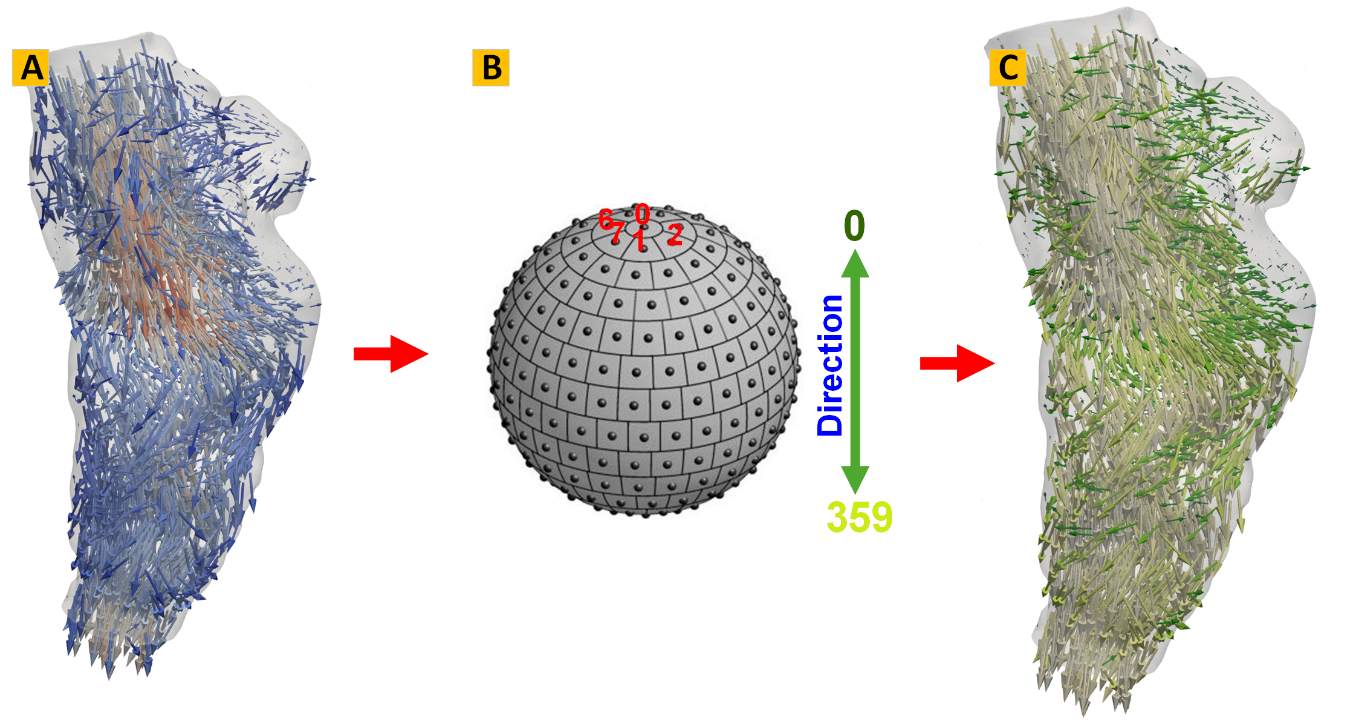


**Supplementary Figure S1**: A graphical illustration of the procedures involved for calculating Directional velocity informatics: (a) isolating velocity vectors within IA, (b) utilizing Leopardi’s method for defining velocity directions (each partition on the unit sphere corresponds to one unique direction, and (c) computed angular directions for every velocity vector. Recall that all velocity vectors were converted to a rectilinear grid; thus, this process yielded 3D DVelocity images.

As illustrated in Figure S1, the process begins with a 3D CFD-simulated velocity field at the peak systole phase in and around an IA. A previously published IA segmentation algorithm [4] is first applied to isolate the vector velocity field within the aneurysm dome. The identified velocity vectors from the unstructured grid are then resampled via interpolation onto a uniform computing grid with a voxel size of 0.2 × 0.2 × 0.2 mm³.

In the second step (Figure S1B), the direction of each velocity vector is mapped onto an equally partitioned unit sphere divided into 360 sections. Specifically, each velocity vector is assigned to the partition zone on the unit sphere that best aligns with its direction. Consequently, all velocity vectors in the uniform grid are linked to one of the 360 partitions (Figure S2B). These velocity associations are then represented as a three-dimensional 8-bit image, referred to as a directional velocity field/image (Figure S1C). A similar technique is applied to convert velocity magnitudes into 8-bit image files for further analysis.

Once the 3D directional and magnitude velocity images are obtained, the open-source Pyradiomics package[5] is used to compute velocity-informatics parameters in the third step. It is important to note that the Pyradiomics package (<https://pyradiomics.readthedocs.io/en/latest/>) is applied to a directional velocity image rather than a radiographic image. The descriptions provided below are adapted from the PyRadiomics documentation for completeness.

**Second-Order Statistics in Velocity Informatics:**

Second-order statistical features pertain to the interrelationships between neighboring voxel intensities and their spatial arrangements. Several methods have been reported to quantify these interrelationships, including the Gray Level Co-occurrence Matrix (GLCM), the Gray Level Run Length Matrix (GLRLM), and the Gray Level Size Zone Matrix (GLSZM). Parameters derived from GLCM, GLRLM, and GLSZM are summarized below.

**The Gray Level Co-occurrence Matrix (GLCM)** quantifies the spatial relationship between pairs of connected voxels based on their intensity values. It is denoted as $P(i,j\mid\delta,\theta),$ where $\delta$ represents the distance between the voxels and $\theta$ represents the angle of their relative orientation. For 2D images, $\theta$ takes on one of four discrete values, increasing to 13 discrete values for 3D images.

Mathematically, the GLCM of an image (or image-like data) with $Nx\times Ny$ dimensions and $Ng$ different intensity levels is computed using the following equation:

${GLCM}_{\delta}^{\theta}\left( i,j \right)=\left| \left\{ \left( \left( r,s \right),\left( t,v \right) \right):I\left( r,s \right)=i, I\left( t,v \right)=j \right\} \right|\forall i,j\in\{1,2,3,\ldots,N_{g}\}$ (1)

Where $(t,v)$ is defined as:

$\left( t,v \right)=\left\{ \begin{aligned} \left( r+\delta,s \right) if \theta=0^{\circ} \\ \left( r+\delta,s+\delta\right) if \theta={45}^{\circ} \\ \left( r,s+\delta\right) if \theta={90}^{\circ} \\ \left( r-\delta,s-\delta\right) if \theta={135}^{\circ} \end{aligned} \right.$ and |. | represents the number of components in a set.

**Supplementary Table S4**: A summary of GLCM variable

| Paramete | Description |
| --- | --- |
| Autocorrelation | Quantifies the magnitude of fineness and coarseness of an image texture and can be estimated as: $Autocorrelation= \sum_{i=1}^{N_{g}} \sum_{j=1}^{N_{g}} p\left( i,j \right)i$ |
| Cluster Shade | Quantifies the level of skewness and uniformity of GLCM, and its higher value implies more significant asymmetry around the average value.  $Cluster Shade = \sum_{i=1}^{N_{g}} \sum_{j=1}^{N_{g}} {(i+j-\mu_{x}-\mu_{y})}^{3}p(i,j)$ |
| Cluster Tendency | Measures sets of voxels with an identical intensity value.  $Cluster Tendency = \sum_{i=1}^{N_{g}} \sum_{j=1}^{N_{g}} {(i+j-\mu_{x}-\mu_{y})}^{2}p(i,j)$ |
| Idm | Quantifies the local homogeneity of an image. It assesses how similar intensity values are within neighboring pixel pairs, with higher values indicating greater homogeneity.  $Idm=\sum_{i=1}^{N_{g}} \sum_{j=1}^{N_{g}} \frac{P_{x-y}(\kappa)}{1+\kappa^{2}}$ |
| Joint Average | Compute mean intensity level i distribution.  $Joint Average=\mu_{x}= \sum_{i=1}^{N_{g}} \sum_{i=1}^{N_{g}} p\left( i,j \right)i$ |
| Joint Entropy | Quantifies the range of randomness in adjacent intensity values.  $Joint Average=\mu_{x}= \sum_{i=1}^{N_{g}} \sum_{j=1}^{N_{g}} p\left( i,j \right)i$ |
| Joint Energy | Estimates the homogeneity of a pattern in the image.  $Joint Energy=\mu_{x}= \sum_{i=1}^{N_{g}} \sum_{j=1}^{N_{g}} {(p\left( i,j \right))}^{2}$ |
| Maximum Probability | Quantifies the number of the most common set of adjacent intensity levels.  $Maximum Probability=max(p\left( i,j \right))$ |
| Sum Average | Estimates the association between the occurrences of connected pixels with lower and higher intensity levels.  $Sum Average=\sum_{\kappa=w}^{2N_{g}} P_{x+y}\left( \kappa\right)\kappa, whereP_{x+y}\left( \kappa\right)=$ $\sum_{i=1}^{N_{g}} \sum_{j=1}^{N_{g}} p\left( i,j \right)$  and $i+j=\kappa, \kappa=2,3, \ldots,2N_{g}$ |
| Sum Entropy | Aggregates of neighborhood intensity values distinctions.  $Sum Entropy= \sum_{i=1}^{N_{g}} \sum_{j=1}^{N_{g}} P_{x+y}(\kappa){log}_{2}(P_{x+y}\left( \kappa\right)+\epsilon)$ |

**The Gray Level** **Run Length Matrix (GLRLM) is** is computed based on the number of connected voxels in the same intensity. GLRLM is characterized by an angle between pairs of voxels, $\theta$. Elements $(i,j)$ in the matrix represents the number of voxels with intensity i and run length j in a specified direction.

Mathematically, the GLRLM of an image with $Nx \times Ny$ dimensions and $Ng$ different intensity levels is computed using the following equation

${GLRM}_{\theta}\left( i,j \right)=\left| \left\{ \left( m,n \right):I\left( k,l \right)\epsilon Nb\left( m,n,j,\theta\right):I\left( k,l \right)=i \right\} \right|\forall i,j\in\{1,2,3,\ldots,N_{g}\}$ (2)

where $Nb\left( m,n,j,\theta\right)=\left\{ \begin{aligned} \{\left( m+1,n \right),\left( m+2,n \right), \ldots,(m+j,n)\} if \theta=0^{\circ} \\ \{\left( m+1,n+1 \right),\left( m+2,n+2 \right), \ldots,(m+j,n+j)\} if \theta={45}^{\circ} \\ \{\left( m,n+1 \right),\left( m,n+2 \right), \ldots,(m,n+j))\} if \theta={90}^{\circ} \\ \{\left( m-1,n-1 \right),\left( m-2,n-2 \right), \ldots,(m-j,n-j)\} if \theta={135}^{\circ} \end{aligned} \right.$

**Supplementary Table S5**: A summary of GLRM variable

| Paramete | Description |
| --- | --- |
| GrayLevelNonuniformity | Measures the similarity of gray-level intensity values within an image. A lower GLN value indicates greater uniformity or similarity in intensity values across the image, implying less variation and a more consistent distribution of gray levels throughout the image.  $GrayLevelNonuniformity=\frac{\sum_{i=1}^{N_{g}} \sum_{j=1}^{N_{r}} p\left( i,j\vert\theta\right)^{2}}{N_{r}\theta}$ |
| HighGrayLevelRunEmphasis | Quantifies the distribution of voxels with higher-intensity values.  $HighGrayLevelRunEmphasis=\frac{\sum_{i=1}^{N_{g}} \sum_{j=1}^{N_{r}} p\left( i,j\vert\theta\right)i^{2}}{N_{r}\theta}$  Where $N_{r}(\theta)$ is a number of runs in an image along angle 𝜃 and calculated as follows: $N_{r}\left( \theta\right)=\sum_{i=1}^{N_{g}} \sum_{j=1}^{N_{r}} p\left( i,j\vert\theta\right), 1\leq N_{r}\left( \theta\right)\leq N_{p}$ |
| LongRunHighGrayLevelEmphasis | Quantifies joint distribution of voxels with higher intensity and long run length.  $LongRunHighGrayLevelEmphasis=\frac{\sum_{i=1}^{N_{g}} \sum_{j=1}^{N_{r}} p\left( i,j\vert\theta\right)i^{2}j^{2}}{N_{r}\theta}$ |
| LongRunLowGraylevelEmphasis | Measures sets of voxels with an identical intensity value.  $LongRunLowGrayLevelEmphasis=\frac{\sum_{i=1}^{N_{g}} \sum_{j=1}^{N_{r}} \frac{p\left( i,j\vert\theta\right)j^{2}}{i^{2}}}{N_{r}\theta}$ |
| LowGrayLevelRunEmphasis | Estimate the distribution of images' lower intensity values $LowGrayLevelEmphasis=\frac{\sum_{i=1}^{N_{g}} \sum_{j=1}^{N_{r}} \frac{p\left( i,j\vert\theta\right)}{i^{2}}}{N_{r}\theta}$ |
| RunVariance | Measures variance of runs based on existing run length.  $RunVariance= \sum_{i=1}^{N_{g}} \sum_{j=1}^{N_{r}} p\left( i,j\vert\theta\right){(j-\mu)}^{2}$  Where $\mu= \sum_{i=1}^{N_{g}} \sum_{j=1}^{N_{r}} p\left( i,j\vert\theta\right)j$ |

**The Gray Level** **Size Zone Matrix (GLSZM)** quantifies intensity zones in an image. A zone is defined as some connected voxels with the same intensity level. The $(i,j)th$ element of GLSZM represents the number of zones with intensity i and size j seen in the image. Unlike the GLCM and GLRLM, there is no dependency on the $\theta$ to the generation of GLSZM, and thus, only a unique matrix will be calculated considering different directions.

**Supplementary Table S6**: A summary of GLSZM variable

| Paramete | Description |
| --- | --- |
| HighGraylevelzoneEmphasis | Measures the distribution of higher intensity size zones.  $HighGraylevelzoneEmphasis=\frac{\sum_{i=1}^{N_{g}} \sum_{j=1}^{N_{s}} p\left( i,j \right)i^{2}}{N_{z}}$ |
| GrayLevelVariance | Measures variance of intensity level based on existing zones.  $GrayLevelVariance= \sum_{i=1}^{N_{g}} \sum_{j=1}^{N_{s}} p\left( i,j\vert\theta\right){(j-\mu)}^{2}$  Where $\mu= \sum_{i=1}^{N_{g}} \sum_{j=1}^{N_{s}} p\left( i,j\vert\theta\right)j$ |
| SizeZoneNonUniformity | Quantifies the variability of size zone volumes within an image.  $SizeZoneNonUniformity= \frac{1}{N_{z}}\sum_{j=1}^{N_{s}} \sum_{i=1}^{N_{g}} p\left( i,j \right)^{2}$ |
| SmallAreaEmphasis | Evaluates the distribution of small-size zones within a texture. Its higher value suggests a higher prevalence of smaller size zones and finer textures, highlighting the presence of intricate details and finer variations in the image texture.  $SmallAreaEmphasis= \frac{1}{N_{z}}\sum_{i=1}^{N_{g}} \sum_{j=1}^{N_{s}} \frac{p\left( i,j \right)}{j^{2}}$ |
| Zone Percentage | Quantifies the texture's coarseness based on the ratio between number of zones and number of voxels.  $ZonePercentage= \frac{N_{z}}{N_{p}}$  Where $N_{z}$ represents the number of zones in ROI and is calculated as $N_{z}=\sum_{i=1}^{N_{g}} \sum_{j=1}^{N_{r}} p\left( i,j \right)$ |
| ZoneEntropy | Quantifies the uncertainty or randomness in the distribution of zone sizes and gray levels.  $ZoneEntropy= \sum_{i=1}^{N_{g}} \sum_{j=1}^{N_{s}} p\left( i,j \right){log}_{2}(p\left( i,j \right)+\varepsilon)$ |
|  |  |

**
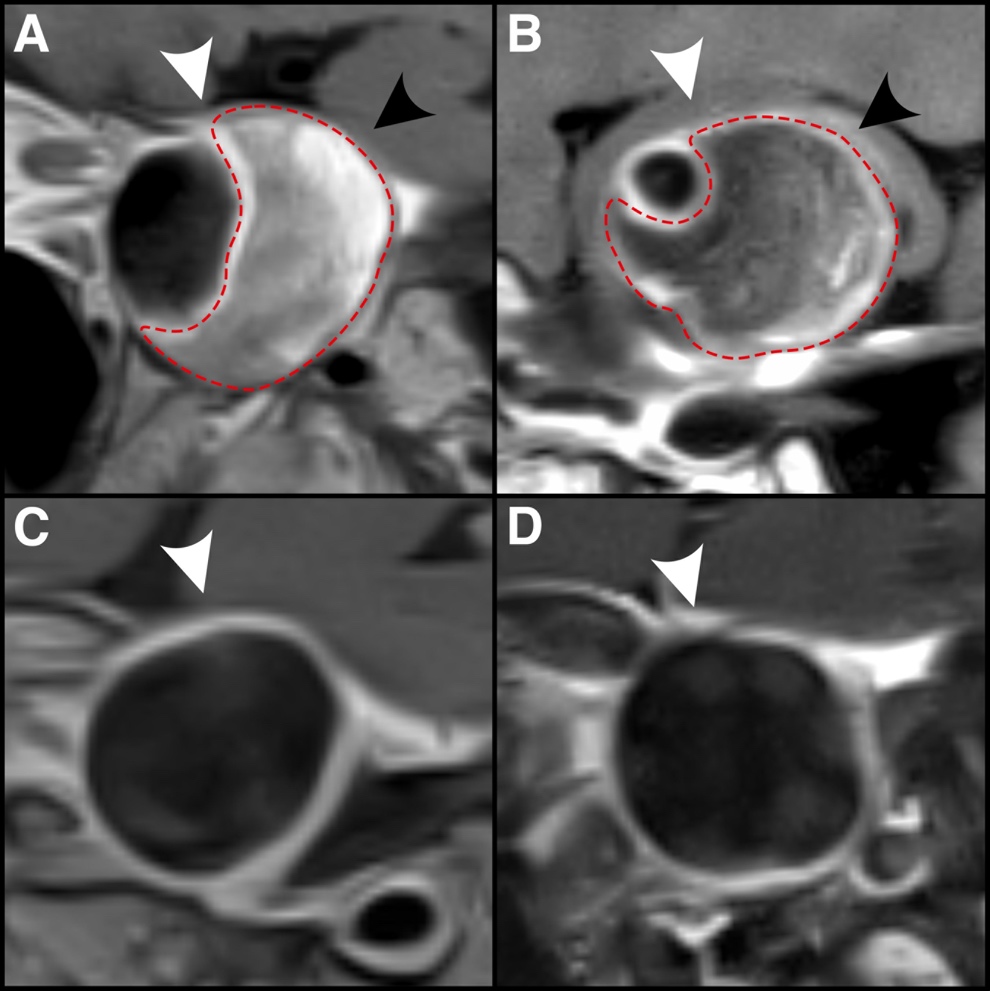
** **Section 5 An Example of High-Resolution MRI Data**

**Supplementary Figure S2:** Thrombosed and Non-Thrombosed Intracranial Aneurysms. Sagittal view of high-resolution magnetic resonance imaging (HR-MRI) shows two intracranial aneurysms (white arrows) located in the cavernous portion of the internal carotid artery (ICA) with 24.5 mm (A) and 23.6 mm (B) in diameter with intrasaccular thrombosis (Black arrow). (B) Two non-thrombosed aneurysms located in the cavernous ICA with 24.2 mm (C) and 23.3 mm (D) in diameter were matched with the previous thrombosed intracranial aneurysms.

**Section 6 STROBE Statement—Checklist of items that should be included in reports of *cross-sectional studies***

|  | Item No | Recommendation | Page No |
| --- | --- | --- | --- |
| **Title and abstract** | 1 | (*a*) Indicate the study’s design with a commonly used term in the title or the abstract | 2 |
|  |  | (*b*) Provide in the abstract an informative and balanced summary of what was done and what was found | 2 |
| Introduction | | | |
| Background/rationale | 2 | Explain the scientific background and rationale for the investigation being reported | 3 |
| Objectives | 3 | State specific objectives, including any prespecified hypotheses | 3 |
| Methods | | | |
| Study design | 4 | Present key elements of study design early in the paper | 3 |
| Setting | 5 | Describe the setting, locations, and relevant dates, including periods of recruitment, exposure, follow-up, and data collection | 3 |
| Participants | 6 | (*a*) Give the eligibility criteria, and the sources and methods of selection of participants | 3 |
| Variables | 7 | Clearly define all outcomes, exposures, predictors, potential confounders, and effect modifiers. Give diagnostic criteria, if applicable | 6-10 |
| Data sources/ measurement | 8* | For each variable of interest, give sources of data and details of methods of assessment (measurement). Describe comparability of assessment methods if there is more than one group | *6-10* |
| Bias | 9 | Describe any efforts to address potential sources of bias | 3 |
| Study size | 10 | Explain how the study size was arrived at | 3 |
| Quantitative variables | 11 | Explain how quantitative variables were handled in the analyses. If applicable, describe which groupings were chosen and why | 5-10 |
| Statistical methods | 12 | (*a*) Describe all statistical methods, including those used to control for confounding | 10 |
|  |  | (*b*) Describe any methods used to examine subgroups and interactions | N/A |
|  |  | (*c*) Explain how missing data were addressed | N/A |
|  |  | (*d*) If applicable, describe analytical methods taking account of sampling strategy | N/A |
|  |  | (*e*) Describe any sensitivity analyses | N/A |
| Results | | | |
| Participants | 13* | (a) Report numbers of individuals at each stage of study—eg numbers potentially eligible, examined for eligibility, confirmed eligible, included in the study, completing follow-up, and analysed | 10 |
|  |  | (b) Give reasons for non-participation at each stage | N/A |
|  |  | (c) Consider use of a flow diagram | N/A |
| Descriptive data | 14* | (a) Give characteristics of study participants (eg demographic, clinical, social) and information on exposures and potential confounders | 11,12 |
|  |  | (b) Indicate number of participants with missing data for each variable of interest | N/A |
| Outcome data | 15* | Report numbers of outcome events or summary measures | 11,12 |
| Main results | 16 | (*a*) Give unadjusted estimates and, if applicable, confounder-adjusted estimates and their precision (eg, 95% confidence interval). Make clear which confounders were adjusted for and why they were included | N/A |
|  |  | (*b*) Report category boundaries when continuous variables were categorized | 11,12 |
|  |  | (*c*) If relevant, consider translating estimates of relative risk into absolute risk for a meaningful time period | N/A |
| Other analyses | 17 | Report other analyses done—eg analyses of subgroups and interactions, and sensitivity analyses | N/A |
| Discussion | | | |
| Key results | 18 | Summarise key results with reference to study objectives | 13 |
| Limitations | 19 | Discuss limitations of the study, taking into account sources of potential bias or imprecision. Discuss both direction and magnitude of any potential bias | 15 |
| Interpretation | 20 | Give a cautious overall interpretation of results considering objectives, limitations, multiplicity of analyses, results from similar studies, and other relevant evidence | 13,14 |
| Generalisability | 21 | Discuss the generalisability (external validity) of the study results | 14 |
| Other information | | | |
| Funding | 22 | Give the source of funding and the role of the funders for the present study and, if applicable, for the original study on which the present article is based | 15 |

*Give information separately for exposed and unexposed groups.

**Note:** An Explanation and Elaboration article discusses each checklist item and gives methodological background and published examples of transparent reporting. The STROBE checklist is best used in conjunction with this article (freely available on the Web sites of PLoS Medicine at http://www.plosmedicine.org/, Annals of Internal Medicine at http://www.annals.org/, and Epidemiology at http://www.epidem.com/). Information on the STROBE Initiative is available at www.strobe-statement.org.

**Reference**

[1] K. Sunderland *et al.* (2021) Quantitative analysis of flow vortices: differentiation of unruptured and ruptured medium-sized middle cerebral artery aneurysms. Acta Neurochirurgica*.*  163: 2339-2349 doi: 10.1007/s00701-020-04616-y.

[2] J. Jiang *et al.* (2023) Augmenting Prediction of Intracranial Aneurysms’ Risk Status Using Velocity-Informatics: Initial Experience. Journal of Cardiovascular Translational Research*.*  doi: 10.1007/s12265-023-10394-6.

[3] J. Jiang *et al.* (2023) Augmenting prediction of intracranial aneurysms’ risk status using velocity-informatics: initial experience. 16: 1153-1165.

[4] J. Jiang and C. M. Strother (2013) Interactive Decomposition and Mapping of Saccular Cerebral Aneurysms Using Harmonic Functions: Its First Application With “Patient-Specific” Computational Fluid Dynamics (CFD) Simulations. IEEE Transactions on Medical Imaging*.*  32: 153-164 doi: 10.1109/TMI.2012.2216542.

[5] J. J. M. van Griethuysen *et al.* (2017) Computational Radiomics System to Decode the Radiographic Phenotype. Cancer Research*.*  77: e104-e107 doi: 10.1158/0008-5472.CAN-17-0339 %J Cancer Research.
